# ROCK Inhibition Suppresses Thrombospondin-1 and Rewires DNA Methylation in the Trabecular Meshwork

**DOI:** 10.3390/ijms27209248

**Published:** 2026-10-18

**Authors:** Sze-wan Shan, Kit-ying Choy, Choi-ying Ling, Hoi-lam Li, Hong-lok Lung, Chi-ho To, Chi-wai Do

**Affiliations:** 1 School of Optometry, The Hong Kong Polytechnic University, Hong Kong, China; kitying.choy@polyu.edu.hk (K.-y.C.);; 2 Centre for Eye and Vision Research (CEVR), 17W Hong Kong Science Park, Hong Kong, China; 3 Research Centre for SHARP Vision (RCSV), The Hong Kong Polytechnic University, Hong Kong, China; 4 Ophthalmology, Boston University Chobanian & Avedisian School of Medicine, Boston, MA 02118, USA; 5 Department of Chemistry, Hong Kong Baptist University, Hong Kong, China; 6 School of Optometry and Vision Sciences, Cardiff University, Cardiff, UK

**Keywords:** ROCK inhibitor, Y39983, THBS1, trabecular meshwork, DNA methylation, DNMT1, DNMT3A, WGBS, focal adhesion

## Abstract

Rho-associated protein kinase (ROCK) inhibitors reduce cytoskeletal tension in trabecular meshwork (TM) cells, but the mechanisms linking this acute response to sustained extracellular matrix remodelling remain incompletely understood. In this study, we investigated whether the ROCK inhibitor Y39983 regulates thrombospondin-1 (THBS1) and DNA methylation programs, elements known to modulate extracellular matrix (ECM) remodelling, in primary human trabecular meshwork (hTM) cells. Primary hTM cells were treated with Y39983, 5-azacytidine (5-Aza, a DNA methyltransferase inhibitor), or both in combination. Gene expression of THBS1, DNA methyltransferase, ECM, and cytoskeletal molecules was assessed using RT-qPCR. *THBS1* promoter methylation was examined through methylation-specific PCR, while genome-wide methylation remodelling was profiled using whole-genome bisulphite sequencing (WGBS) followed by pathway enrichment analysis. 5-Azacytidine abolished Y39983-dependent effects on DNMT1 and DNMT3A and attenuated *THBS1* regulation. Methylation-specific PCR suggested altered *THBS1* promoter methylation after Y39983 treatment, while whole-genome bisulphite sequencing identified 8845 differentially methylated genes, including 5608 hypomethylated and 3237 hypermethylated genes, enriched in ErbB signalling, focal adhesion, and regulation of the actin cytoskeleton. Independently, Y39983 reduced the basal and stimulus-induced expression of *THBS1*, as well as ECM- and cytoskeleton-associated genes, including *COL4A1, COL6A1*, and *ACTG1*. These findings demonstrate that ROCK inhibition is associated with changes in DNMT-related gene expression and DNA methylation patterns in hTM cells. However, because 5-azacytidine has effects beyond selective DNMT inhibition, further studies using orthogonal approaches are required to establish whether DNA methylation directly mediates transcriptional responses to Y-39983.

## 1. Introduction

Glaucoma is a leading cause of irreversible blindness worldwide and is characterised by progressive optic neuropathy, which is strongly associated with elevated intraocular pressure (IOP). The primary determinant of IOP in most patients is resistance to aqueous humor drainage through the conventional outflow pathway, which consists of the trabecular meshwork (TM), juxtacanalicular tissue (JCT), and Schlemm’s canal. Increased outflow resistance in glaucoma is closely linked to aberrant cytoskeletal tension, altered cell–matrix adhesion, and excessive extracellular matrix (ECM) deposition within the TM, particularly in the JCT, which is considered the major site of outflow resistance [1]. Consequently, targeting the cellular and molecular mechanisms that regulate TM contractility and ECM turnover has emerged as an important therapeutic strategy [2,3].

Rho-associated coiled-coil-containing protein kinases (ROCK1 and ROCK2) are central regulators of actin cytoskeletal organisation, controlling myosin light-chain phosphorylation, stress fibre formation, and focal adhesion maturation downstream of RhoA signalling [4]. The inhibition of ROCK activity reduces actomyosin-driven contractility and promotes the relaxation of TM cells, leading to decreased tissue stiffness and enhanced aqueous humor outflow [4]. Pharmacological ROCK inhibitors have demonstrated clinical efficacy in lowering IOP, validating the TM as a therapeutically tractable target [3,4,5]. At the cellular level, ROCK inhibition induces the disassembly of actin stress fibres and focal adhesions and reverses pathological TM contraction in both two-dimensional cultures and three-dimensional ECM environments, highlighting the importance of cytoskeletal remodelling in restoring outflow function [1,6].

Despite these advances, the molecular mechanisms linking acute ROCK inhibition to longer-term changes in ECM composition and adhesion networks remain incompletely understood. While short-term responses are driven by rapid cytoskeletal relaxation, the sustained modulation of outflow resistance likely requires transcriptional and epigenetic reprogramming that stabilises ECM remodelling and cell–matrix interactions [2]. In particular, the relationship between ROCK signalling and profibrotic pathways, most notably transforming growth factor-β (TGFβ), has received considerable attention, as TGFβ signalling promotes ECM accumulation, increased tissue stiffness, and elevated outflow resistance in glaucoma [7]. However, the exact pathway(s) remain elusive. Therefore, identifying key mediators that couple ROCK-dependent cytoskeletal changes to ECM and growth factor signalling is crucial for understanding how ROCK inhibitors achieve sustained therapeutic effects.

Thrombospondin-1 (THBS1) is a matricellular ECM protein that regulates multiple aspects of cell behaviour, including adhesion, migration, and ECM assembly [8]. Unlike structural ECM proteins, THBS1 functions primarily as a signalling modulator, interacting with cell-surface receptors and growth factors to modulate matrix organisation and cell–matrix communication [8]. In the TM, THBS1 is localised to extracellular regions of the juxtacanalicular tissue and is upregulated in response to TGFβ and glucocorticoid exposure, both of which are strongly implicated in glaucoma pathogenesis. Importantly, THBS1 can activate latent TGFβ complexes through specific peptide interactions, thereby amplifying profibrotic signalling in the extracellular environment [9,10]. This functional linkage positions THBS1 as a putative intermediary, connecting ECM remodelling with growth factor activation [9].

Epigenetic regulation, especially DNA methylation, may provide the missing link between transient ROCK inhibition and durable transcriptional change. DNA methylation, mediated by DNA methyltransferases (DNMTs), can integrate extracellular signals into stable alterations in gene expression and has been implicated in fibrosis, mechano-transduction, and cellular stress responses [11,12]. In the TM, altered DNA methylation has been associated with profibrotic gene activation, ECM deposition, and glucocorticoid-induced dysfunction [2]. Previous work has shown that glucocorticoids can alter THBS1 promoter methylation and DNMT expression in TM cells [13], while ROCK inhibition suppresses THBS1 expression [14], suggesting that THBS1 may serve as an important mediator linking mechano-transduction and epigenetic regulation in the TM. This raises the possibility that ROCK inhibitors regulate TM physiology not only through acute cytoskeletal relaxation, but also through methylation-dependent transcriptional reprogramming [2,12]. Therefore, THBS1 was selected as a candidate target for focused mechanistic investigation in the present study.

The principal contribution of the current study is the proposed association between ROCK inhibition and DNA methylation/epigenetic remodelling in primary hTM cells. It was hypothesised that Y39983 treatment may be associated with methylation-related and transcriptional changes. DNMT expression and 5-azacytidine experiments assessed the involvement of methylation-related processes; MSP examined methylation at *THBS1*; and WGBS characterised genome-wide methylation changes. Pathway analysis identified the biological processes that were enriched among differentially methylated genes, while expression studies evaluated the corresponding responses of selected ECM- and cytoskeleton-associated genes. Together, these complementary approaches were used to examine whether ROCK inhibition is associated with coordinated epigenetic and transcriptional remodelling, without establishing a causal relationship among DNMT activity, DNA methylation, and gene expression.

## 2. Results

### 2.1. 5-Azacytidine Modifies Y39983-Associated Changes in DNMT1, DNMT3A, and THBS1 Transcript Levels

**Figure 1:**
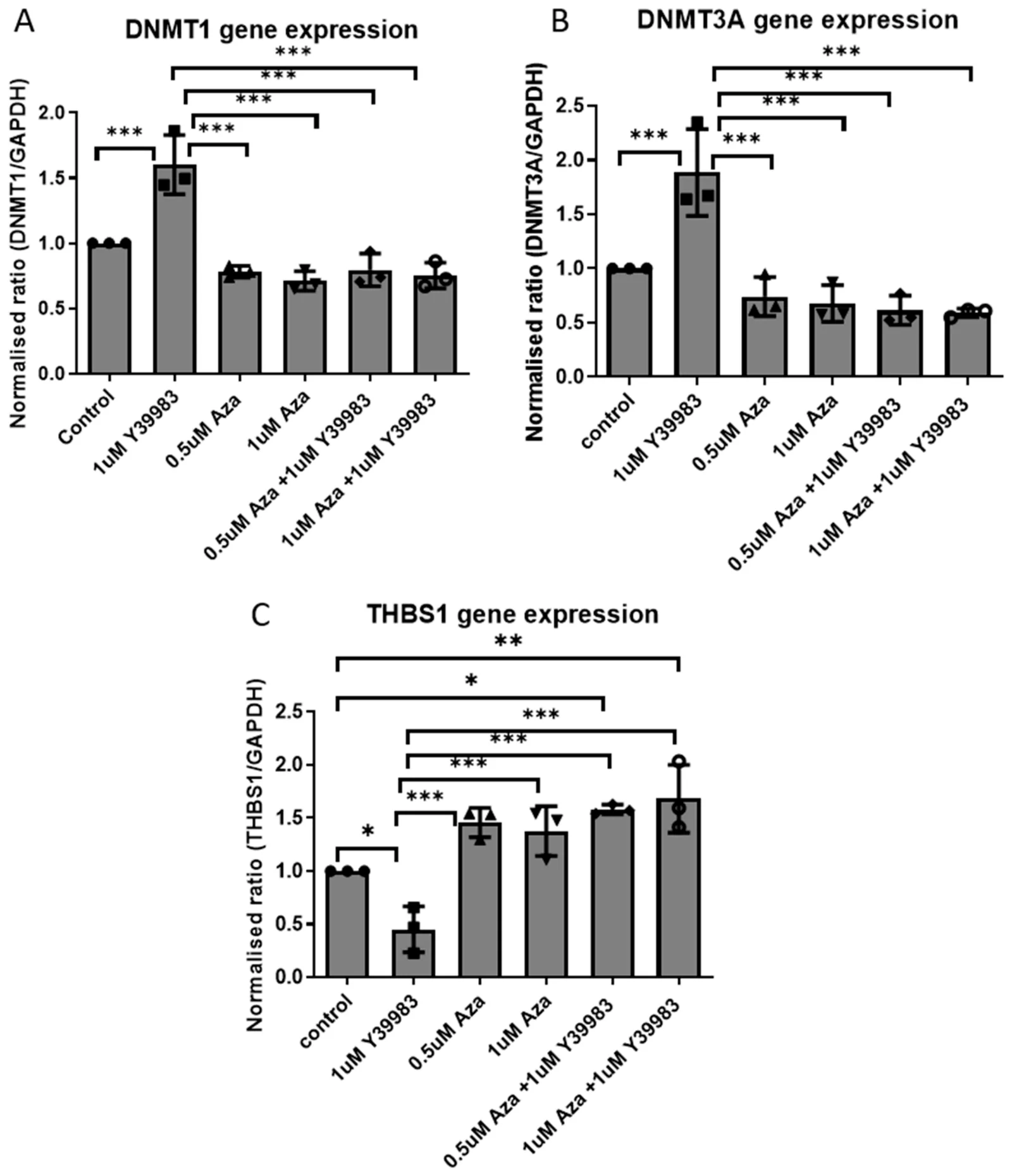
DNA methylation inhibition abolished Y39983-mediated effects on DNMT1/3A and altered THBS1 regulation. Three individual biological replicates of hTM cells were treated with vehicle control, Y39983 (1 µM), 5-azacytidine (5-Aza; 0.5 or 1 µM), or a combination of Y39983 and 5-Aza. (**A**) *DNMT1* expression levels and (**B**) *DNMT3A* expression levels, showing similar regulatory patterns. (**C**) *THBS1* expression in response to treatments, demonstrating attenuation of Y39983-induced regulation by 5-Aza; 5-Aza alone increased *THBS1* expression. Co-treatment with 5-Aza abolished the effects of Y39983 on *DNMT1* and *DNMT3A* expression and modified *THBS1* regulation. Data are presented as mean ± SD. Statistical analysis was performed using one-way ANOVA followed by appropriate post hoc tests; * *p* < 0.05; ** *p* < 0.01; *** *p* < 0.001.

hTM cells were treated with vehicle, Y39983 (1 µM), 5-azacytidine (5-Aza; 0.5 or 1 µM), or Y39983 + 5-Aza (0.5 or 1 µM). While the Y39983 treatment was found to be associated with elevated *DNMT1/3A* gene expression and a concomitant downregulation of *THBS1*, co-treatment with 5-azacytidine prevented the Y39983-induced increase in *DNMT1* and *DNMT3A* expression (Figure 1A,B) and attenuated *THBS1* regulation (Figure 1C).

### 2.2. MSP Suggests Altered THBS1 Promoter Methylation Patterns After Y39983

**Figure 2:**
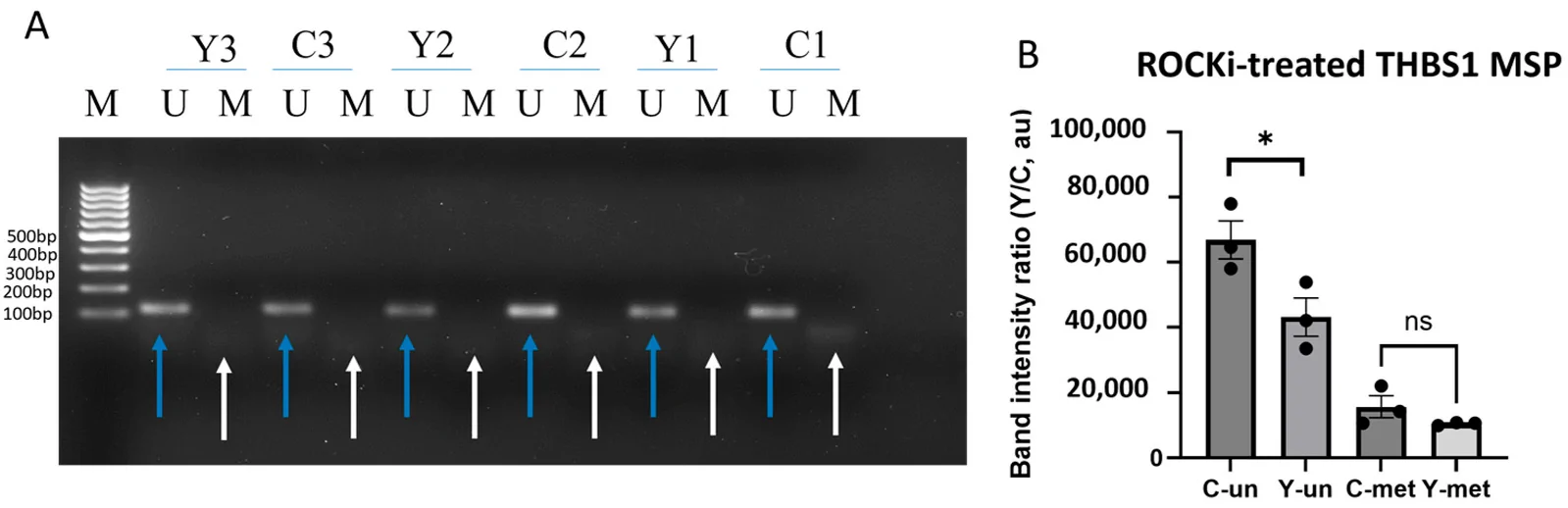
MSP signal patterns of the THBS1 promoter following Y39983 treatment. Methylation-specific PCR was performed using three independent biological replicates of hTM cells treated with vehicle control (C) or Y39983 (Y). (**A**) Both methylated (M, white arrow) (115bp) and unmethylated (U, blue arrow) (75bp) PCR products were detected. Unmethylated THBS1 MSP signals were stronger in control samples than in the corresponding Y39983-treated samples (Y1, Y2, and Y3), whereas methylated signals were not consistently different between groups. The observed differences reflect changes in MSP signal intensity and should not be interpreted as conclusive evidence of altered THBS1 promoter methylation. (**B**) Relative MSP signal intensities were quantified across three biological replicates. Bars represent the mean ± standard deviation, and black circles indicate individual biological replicates. Statistical analysis was performed using Student’s paired *t*-test. *n* = 3 per group. ns, not significant; * *p* < 0.05.

MSP analysis of the *THBS1* promoter showed both methylated and unmethylated products in control cultures (Figure 2). In two donor strains, control cultures exhibited higher unmethylated *THBS1* signals than Y39983-treated cultures, whilst the methylated signal was not consistently different. These results suggest a difference in the unmethylated signal within the primer-defined THBS1 promoter region following Y39983 treatment. However, because the methylated signal was not consistently different among donor strains and MSP is a targeted semiquantitative assay, these findings should be regarded as preliminary and require confirmation through quantitative locus-specific methylation analysis.

### 2.3. Exploratory WGBS Analysis Identifies Candidate Methylation Changes in Genes Associated with ErbB and Focal Adhesion Pathways

**Figure 3:**
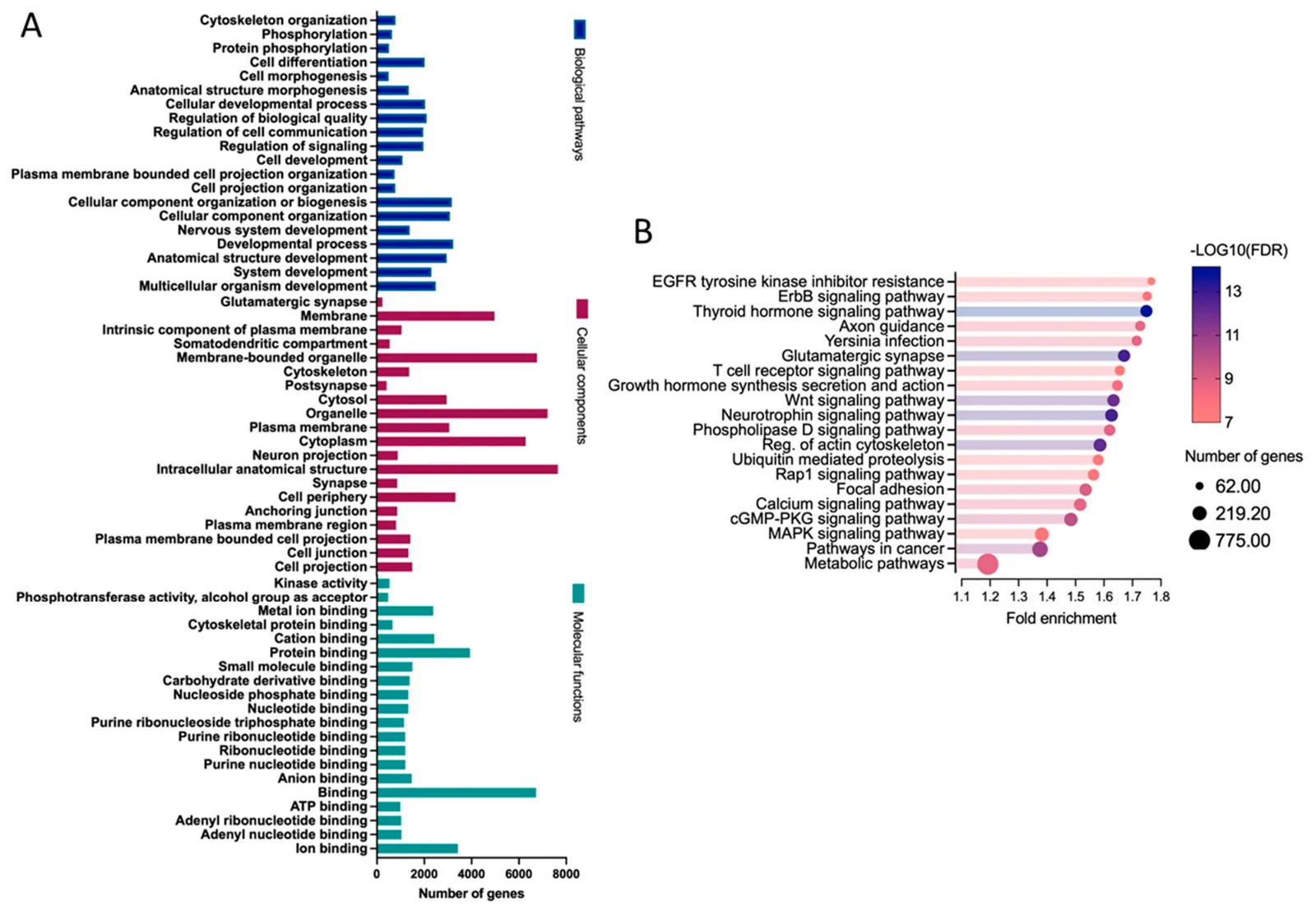
Whole-genome bisulphite sequencing (WGBS) revealed pathway-enriched DNA methylation remodelling in Y39983-treated hTM cells. WGBS was performed to compare DNA methylation profiles between vehicle-treated and Y39983-treated hTM cells. Differentially methylated genes (DMGs) were identified using β-values and Δβ (vehicle−treated) with *p* < 0.05. A total of 8845 DMGs were detected, including 5608 hypomethylated and 3237 hypermethylated genes. (**A**) Network and functional clustering analysis of enriched pathways illustrating interactions among DMGs, with prominent modules linking focal adhesion and regulation of the actin cytoskeleton. The network visualisation demonstrates coordinated epigenetic remodelling in pathways relevant to cytoskeletal dynamics and cell adhesion following Y39983 treatment. (**B**) KEGG pathway enrichment analysis of DMGs showing significantly enriched signalling pathways, including ErbB signalling and EGFR tyrosine kinase inhibitor resistance. Pathways are ranked based on enrichment significance (FDR < 0.05), highlighting functional biological processes associated with Y39983-induced methylation changes.

WGBS compared paired vehicle-control and Y39983-treated hTM cell cultures derived from the same three independent hTM donor strains. Differentially methylated genes (DMGs) were defined using β-values and absolute values of delta beta ≥ 0.1 AND *p* < 0.05 and analysed for Gene Ontology (GO) and Kyoto Encyclopedia of Genes and Genomes (KEGG) enrichment using ShinyGO (v0.85) (a graphical tool for enrichment analysis) with FDR (false discovery rate analysis) < 0.05 (Figure 3A,B). A total of 8845 DMGs were identified (5608 hypomethylated; 3237 hypermethylated). KEGG enrichment highlighted ErbB signalling and EGFR tyrosine kinase inhibitor resistance, while Pathview visualisation further demonstrated that DMGs were broadly distributed across key KEGG pathways, identified by enrichment analysis. In the focal adhesion pathway, DMGs were mapped to extracellular matrix–integrin signalling, focal adhesion kinase signalling, actin cytoskeleton regulation, and PI3K-Akt/MAPK-related components (Supplementary Figure S1). In the ErbB signalling pathway, DMGs were detected across ErbB receptor modules and downstream RAS-RAF-MEK-ERK, PI3K-AKT-mTOR, Src/FAK, and STAT-associated signalling axes (Supplementary Figure S2). These findings suggest that Y39983 treatment is associated with widespread DNA methylation changes in genes linked to cell adhesion, cytoskeletal dynamics, receptor tyrosine kinase signalling, and cell survival pathways in hTM cells.

*THBS1* was not included in the BGI-supplied candidate DMG list, and no *THBS1*-associated region met the WGBS DMR reporting criteria. Therefore, the exploratory WGBS analysis did not independently support a qualifying regional methylation difference at the *THBS1* locus. Overall, while the exploratory analysis generated candidate methylation-associated genes and pathways for further investigation, it does not establish statistically validated genome-wide differential methylation following Y39983 treatment.

### 2.4. ROCK Inhibition Suppresses Basal and Stimulus-Induced Upregulation of Fibrotic and Cytoskeletal Genes Identified in the WGBS

COL4A1, COL6A1, and ACTG1 were included in the exploratory WGBS candidate-gene set based on the prespecified Δβ and nominal *p*-value criteria. Because the initial genome-wide analysis was not corrected for multiple testing, their methylation differences should be considered candidate observations rather than statistically validated gene-specific findings. These genes were selected for expression analysis because of their roles in ECM organisation and cytoskeletal regulation and because they occurred within candidate focal adhesion and actin cytoskeleton pathway modules. This may be important, as they are ECM/cytoskeletal components relevant to TM contractility and occur within the exploratory WGBS candidate list of enriched focal adhesion/actin cytoskeleton modules. *THBS1* was included in subsequent expression analyses because of its established role in TM fibrosis and its regulation by Y39983 observed in the MSP experiment and a previous gene expression study of this study group [14]. Relative gene expression of the identified targets was compared across control-normalised conditions: Y39983, DEX, DEX + Y39983, *THBS1* overexpression, *THBS1* overexpression + Y39983, TGFβ1, and TGFβ1 + Y39983. Across all transcripts, Y39983 alone reduced basal gene expression relative to the control. This suppression was most evident for *THBS1, COL4A1*, and *COL6A1*, which decreased to approximately 0.5–0.7-fold of the control, while *ACTG1* showed a more modest reduction to around 0.7-fold. These results suggest that Y39983 suppresses basal ECM-related and cytoskeletal gene expression under unstimulated conditions.

**Figure 4:**
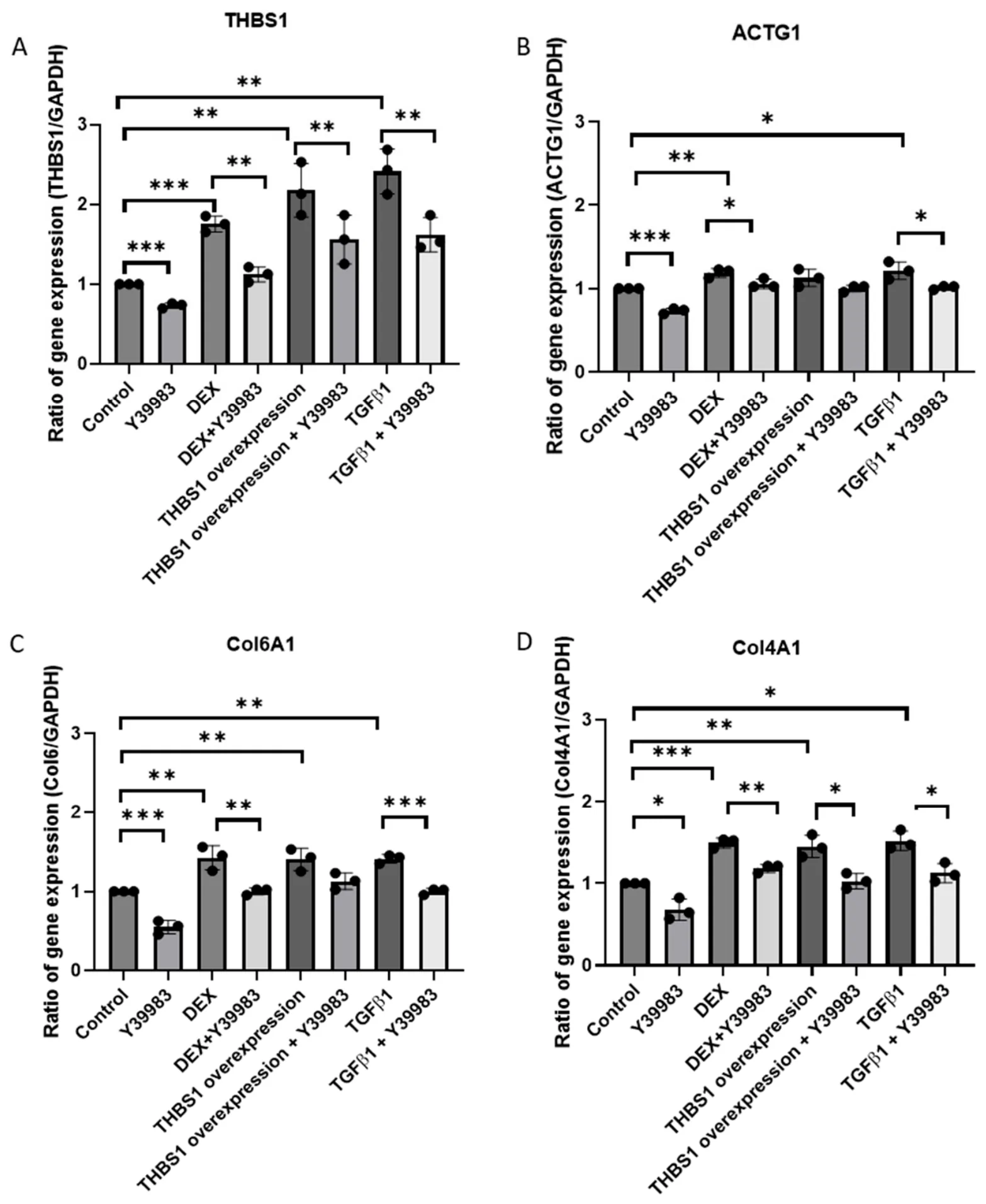
Y39983 suppressed basal and stimulus-induced expression of ECM- and cytoskeleton-associated genes. Relative gene expression levels of (**A**) *THBS1,* (**B**) *COL4A1*, (**C**) *COL6A1*, and (**D**) *ACTG1* were assessed under eight conditions: control, Y39983, DEX, DEX + Y39983, THBS1 overexpression, THBS1 overexpression + Y39983, TGFβ1, and TGFβ1 + Y39983. Gene expression values were normalised to the control group, which was set to 1.0. One-way ANOVA followed by appropriate post hoc tests was used; * *p* < 0.05, ** *p* < 0.01, and *** *p* < 0.001. Data are presented as mean ± SD. Three individual biological replicates of hTM cells were used.

Dexamethasone (DEX) increased the expression of all measured genes, with the strongest induction observed for *THBS1* at approximately 1.8–2.0-fold of the control (Figure 4A). *COL4A1* and *COL6A1* increased expression by about 1.4–1.6-fold (Figure 4C,D), while *ACTG1* (Figure 4B) showed only a modest increase of approximately 1.2-fold. Co-treatment with Y39983 attenuated the DEX-induced response, reducing ECM marker expression towards the baseline and keeping *ACTG1* close to control levels. *THBS1* overexpression increased ECM-associated gene expression, raising *THBS1* approximately 1.7–1.9-fold and *COL4A1/COL6A1* around 1.4–1.6-fold relative to the control. *ACTG1* remained only slightly elevated at about 1.1-fold (Figure 4). The addition of Y39983 reduced these *THBS1* overexpression-associated increases (Figure 4A), particularly for ECM-related genes, bringing expression closer to baseline. TGFβ1 also promoted profibrotic/ECM gene expression, increasing *THBS1, COL4A1,* and *COL6A1* to approximately 1.5–1.9-fold of the control, while *ACTG1* showed a smaller response (Figure 4B). Co-treatment with Y39983 reduced TGFβ1-induced ECM gene expression to about 1.1–1.2-fold, indicating strong suppression of TGFβ1-mediated gene expression.

Overall, these results indicate that DEX-induced TM changes may involve *THBS1* overexpression and TGFβ-related enhancement of ECM-associated gene expression, especially *THBS1, COL4A1,* and *COL6A1*, whereas *ACTG1* responded more modestly. In contrast, Y39983 suppressed basal expression and attenuated stimulus-induced upregulation, supporting its role in dampening ECM gene activation.

## 3. Discussion

Our study provides evidence that short-term ROCK inhibition is associated with multiple molecular changes in primary human TM cells. Following 48 h of Y39983 treatment, changes were observed in THBS1 and selected extracellular matrix- and cytoskeleton-associated transcripts, together with alterations in THBS1 promoter methylation and genome-wide DNA methylation patterns. These concurrent findings suggest that THBS1 may be involved in the molecular response to ROCK inhibition and that methylation-related processes may accompany the observed transcriptional changes. However, the present experiments do not establish a causal relationship between DNA methylation and gene expression, nor do they determine whether these molecular changes persist after treatment withdrawal or produce lasting effects on TM function.

Consistent with prior studies, ROCK inhibition with Y39983 significantly reduced THBS1 transcription, reinforcing the role of ROCK signalling in profibrotic gene regulation [15]. Changes in MSP signal intensity were also observed, although their relationship to THBS1 promoter methylation remains inconclusive. THBS1 is a well-established activator of latent TGFβ and acts as a critical amplifier of extracellular profibrotic signalling [9]. Its suppression therefore provides a plausible mechanism by which ROCK inhibitors attenuate ECM deposition and tissue stiffening, two hallmarks of glaucomatous TM dysfunction [3]. Importantly, the present study extends this framework by demonstrating that ROCK inhibition not only reduces basal *THBS1* expression, but also effectively blunts its induction by glucocorticoids and TGFβ, which are major pathogenic drivers implicated in primary open-angle and steroid-induced glaucoma [2]. These findings position THBS1 as a key convergence point through which Y39983 attenuates the transcriptional upregulation of the selected ECM-associated genes.

Treatment with 5-azacytidine modified the Y39983-associated changes in *DNMT1*, *DNMT3A,* and *THBS1* transcript levels. These findings are consistent with, but do not establish, the involvement of DNA methylation-related regulation in response to ROCK inhibition. Importantly, 5-azacytidine is not selective for *DNMT1* and can affect cellular processes through its incorporation into both DNA and RNA. Consequently, the response to 5-azacytidine cannot be interpreted as definitive evidence that DNMT inhibition or DNA demethylation directly mediates the effects of Y39983. In addition, the present measurements of *DNMT1* and *DNMT3A* mRNA do not establish corresponding changes in DNMT protein abundance or catalytic activity. Although the MSP and WGBS findings independently demonstrate that Y39983 treatment is associated with altered DNA methylation patterns, causal relationships among ROCK inhibition, DNMT activity, methylation changes, and target-gene expression remain to be established. Future studies using additional DNMT inhibitors, DNMT-specific knockdown or genetic manipulation, quantitative locus-specific methylation analysis, and direct DNMT activity measurements are required to define the underlying mechanism.

Genome-wide methylation profiling further revealed extensive epigenetic reprogramming, with 8845 differentially methylated genes significantly enriched in pathways related to ErbB signalling and focal adhesion/actin cytoskeleton regulation, findings which should consequently be regarded as hypothesis-generating. These pathways are highly relevant to TM biology, governing cell–matrix adhesion, mechano-transduction, and cytoskeletal tension [16,17]. The convergence of methylation changes on these functional modules suggests that ROCK inhibition orchestrates coordinated remodelling of both structural and signalling networks. Notably, the enrichment of ErbB signalling is particularly intriguing given its emerging role in fibrotic regulation and its potential crosstalk with integrin-mediated adhesion pathways.

Functionally, these epigenetic changes were associated with a downregulation of key ECM and cytoskeletal genes, including *COL4A1, COL6A1,* and *ACTG1.* ROCK inhibition not only reduced basal expression of these genes, but also suppressed their induction by *THBS1* overexpression, dexamethasone (DEX), and TGFβ. The strong attenuation of TGFβ-driven transcriptional responses is particularly significant given the central role of TGFβ signalling in TM fibrosis and increased outflow resistance [7,9]. Together, these data support a model in which ROCK signalling operates upstream of both cytoskeletal organisation and profibrotic transcriptional programs, with epigenetic modifications acting to consolidate and stabilise these changes over time.

From a therapeutic perspective, the observed suppression of THBS1 and selected extracellular matrix-associated transcripts provides a potential molecular context for the cellular response to ROCK inhibition. Nevertheless, the present study was not designed to evaluate therapeutic durability or disease modification. The experiments did not assess whether the methylation or transcriptional changes persisted after Y39983 withdrawal, and no long-term measurements of trabecular meshwork function, aqueous outflow, or intraocular pressure were performed. Therefore, the clinical and functional relevance of these short-term molecular changes remains to be determined.

These insights have several important clinical implications. First, the ability of ROCK inhibition to suppress *THBS1* and disrupt the feed-forward activation of TGFβ signalling indicates that these agents may actively attenuate fibrotic remodelling of the TM, potentially slowing disease progression in glaucoma [9,18]. This shifts the therapeutic paradigm from purely pressure-lowering strategies toward approaches that address the underlying tissue pathology. Second, the observed suppression of glucocorticoid- and TGFβ-induced gene expression suggests a role of ROCK inhibitors in preventing or mitigating secondary TM dysfunction, including steroid-induced ocular hypertension [9,19].

Although the observed DNA methylation changes raise the possibility that methylation-related processes participate in the cellular response to ROCK inhibition, the present data do not establish DNA methylation as a causal mediator of the transcriptional effects of Y39983. In addition, because persistence after treatment withdrawal was not examined, no conclusions can be drawn regarding epigenetic memory or the durability of these changes. Future studies incorporating treatment-withdrawal experiments, longitudinal methylation and gene expression analyses, DNMT-specific genetic or pharmacological approaches, and functional assessment of trabecular meshwork outflow are needed to address these questions.

The genome-wide enrichment of methylation changes in ErbB and focal adhesion pathways further highlights the broad systems-level impact of ROCK inhibition. Rather than acting as a purely mechanical relaxant, ROCK inhibitors appear to function as integrative modulators of TM biology. These functions suggest that ROCK inhibitors are suited for the treatment of complex, multifactorial diseases such as glaucoma.

Finally, Y39983 suppresses THBS1 and selected extracellular matrix- and cytoskeleton-associated transcripts in primary human TM cells and is associated with alterations in THBS1 promoter methylation and genome-wide DNA methylation patterns. The modification of Y39983-associated transcriptional responses by 5′-azacytidine is consistent with the possible involvement of methylation-related regulatory processes. However, because 5′-azacytidine has broad effects, and the present experiments did not directly manipulate individual DNMTs or specific methylation sites, these findings do not establish a DNMT-dependent or DNA methylation-mediated mechanism. Moreover, the short treatment duration and absence of treatment-withdrawal experiments preclude conclusions regarding the persistence, functional durability, or disease-modifying significance of the observed changes. Orthogonal mechanistic approaches and longitudinal functional studies will be necessary to address these questions.

## 4. Materials and Methods

### 4.1. Primary hTM Cell Culture

Human trabecular meshwork (hTM) cells were isolated as described previously [20,21] and authenticated according to published consensus recommendations [21]. Cells were plated and maintained at 37 °C in low-glucose DMEM (Invitrogen, Carlsbad, CA, USA, Cat# 11885076) supplemented with 10% FBS (Invitrogen, Cat# 10082147) until confluent. Before the experiments were performed, confluent cultures were switched to low-glucose DMEM containing 1% FBS and maintained for at least one week. For combined treatments, confluent hTM cell monolayers were first exposed to DEX (300 nM; Sigma-Aldrich, St. Louis, MO, USA) for 5 days, followed by the addition of Y39983 (1 µM; MedChem Express, Monmouth Junction, NJ, USA) for the final two days, while DEX treatment was continued (seven days total).

For TGFβ1 experiments, cells received recombinant TGFβ1 protein (10 ng/mL; Abcam, Cat# ab50036) for three days, with 1 µM Y39983 added during the final two days, while TGFβ1 was maintained.

For the 5-Aza treatment, 0.5–1 µM (Sigma, Cat# A3656) was used to treat the hTM cells for two days. For THBS1 overexpression experiments, cells were transfected with a human THBS1 ORF mammalian expression plasmid (2 µg; Sino Biological Inc., Beijing, China) for 24 h and then treated with 1 µM Y39983 for 48 h.

Vehicle controls consisted of DMSO for Y39983 and 5-Aza; PBS or assay buffer for DEX and recombinant TGFβ1; and transfection reagent-only controls for THBS1 overexpression. Cells were collected after treatment for downstream assays.

### 4.2. Methylation-Specific PCR

Genomic DNA was isolated from hTM cells using the DNeasy Blood and Tissue Kit (Qiagen, Venlo, The Netherlands, Cat# 69504), followed by bisulphite conversion with the EZ DNA Methylation Bisulphite Kit (Zymo Research, Irvine, CA, USA, Cat# D5001), according to the manufacturers’ instructions. The methylation status of the *THBS1* promoter was assessed using methylation-specific primers targeting methylated *THBS1* sequences—forward, 5′-GCGAGCGTTTTTTTAAAAGTGC-3′, and reverse, 5′-TAAACTCGCAAACCAACTCG-3′—and unmethylated *THBS1* sequences—forward, 5′-GAATGTGAGTGTTTTTTTAAAAGTGTG-3′, and reverse, 5′-CCTAAACTCACAAACCAACTCAA-3′. These primers were originally designed and reported by Herrera-Goepfert et al. [22]. Bisulphite-converted DNA was amplified using the Premix Taq HS Kit (Kusatsu., Shiga, Japan, Cat# R028A). PCR was performed under the following cycling conditions: initial denaturation at 95 °C for 12 min; 45 cycles of denaturation at 95 °C for 30 s, annealing at 55 °C for 30 s, and extension at 72 °C for 30 s; and a final extension at 72 °C for 10 min. PCR products were separated on 2% agarose gels and visualised under UV illumination.

### 4.3. RT-qPCR

The total RNA of hTM cells was extracted and quantified using the RNeasy Micro Kit (Qiagen, Cat# 74004). Reverse transcription of mRNA to cDNA was performed using the High-Capacity cDNA Reverse Transcription Kit (Thermo Fisher Scientific, Waltham, MA, USA, Cat# 4368813), followed by qPCR using the LightCycler 480 SYBR Green I Master (Roche Applied Science, Penzberg, Germany, Cat# 100099593) with primers specific for the target genes: *COL6A1* (forward: 5′-CGACTGCGCTATCAAGAAGG-3′; reverse: 5′-CCACCGAGAAGACTTTGACG-3′), *COL4A1* (forward: 5′-TGAAGGGTGATCCAGGTGAG-3′; reverse: 5′-GACCTGGTGGTCCGGTAAAT-3′), *ACTG1* (forward: 5′-ACGTGTCAGGGCTGAGTGTT-3′; reverse: 5′-CAAGTAACAGCCCACGGTGT-3′), *DNMT1* (forward: 5′-AGACTACGCGAGATTCGAGTC-3′; reverse: 5′-TTGGTGGCTGAGTAGTAGAGG-3′), *DNMT3A* (forward: 5′-GCGGCGAGAGGACTGGCC-3′; reverse: 5′-CGGTCCACCTGAATGCCCAA-3′), *THBS1* (forward: 5′-CGTCCTGTTCCTGATGCATG-3′; reverse: 5′-CCAGGAGAGCTTCTTCCACA-3′), and the internal reference gene, *GAPDH* (forward: 5′-GATTTGGTCGTATTGGGCGC-3′; reverse: 5′-TGGACTCCACGACGTACTCA-3′). qPCR was performed in 96-well plates on the ROCHE LightCycler 480 (Roche Applied Science, Penzberg, Germany). Each 10 μL reaction contained 1× Taq PCR Master Mix, 2 μL of cDNA template, and 1 μM each of forward and reverse primers. The PCR cycling conditions were as follows: initial denaturation at 95 °C for 5 min, followed by 40 cycles of denaturation at 95 °C for 30 s, annealing at 61 °C for 30 s, and extension at 72 °C for 30 s. Melting-curve analysis was performed to confirm amplification specificity and exclude primer–dimer formation. A no-template negative control was included on each plate. Data were analysed using LightCycler software 3.5 (RRID:SCR_012155).

### 4.4. Whole-Genome Bisulphite Sequencing

WGBS was performed using three independent primary hTM donor strains per condition. Vehicle-control and Y39983-treated samples were generated from the same donor strains using a paired experimental design. Donor identity was retained during the analysis, and donor-associated variability was handled through paired statistical testing. Genomic DNA was extracted from hTM cells using a DNeasy Blood and Tissue kit following the manufacturer’s instructions, quantified by UV spectroscopy, and then subjected to WGBS analysis (Beijing Genomics Institute (BGI)). In brief, BGI first constructed the DNA library by fragmenting DNA through sonication with a Bioruptor (Diagenode, Seraing, Belgium), followed by blunt-end formation and methylated adaptor ligation. The ligation products were bisulphite-converted and separated on a 2% TAE agarose gel. Fragments were excised from the agarose gels and purified, followed by PCR amplification and cyclization. Finally, the DNA libraries were sequenced on the DNBseq PE100 platform and raw data were filtered to remove adaptor sequences, contamination, and low-quality reads. The obtained clean data were mapped to the reference genome using the BSMAP software 2.9 (RRID:SCR_005671) [23], followed by the removal of duplicate reads and merging of the mapped results of each library.

The difference in the rate of cytosine methylation (mCG, mCHG, mCHH) for a given DNA region was calculated as the β-value and compared between Y39983-treated and vehicle-control hTM cells. β-value is the ratio of the number of reads covering a methylated cytosine to the total number of reads covering that cytosine. Δβ is defined as the average β-value of vehicle-control samples minus that of Y39983-treated samples. Putative DMRs were identified using a sliding-window approach based on a minimum of five cytosine sites, a two-fold difference in methylation level, and a nominal Fisher’s exact-test threshold of *p* ≤ 0.05. Multiple-testing-adjusted *p*-values and FDR/q-values were not available in the vendor-provided DMR output; accordingly, DMRs were defined using the nominal *p*-value threshold. Final DMRs were subsequently mapped to genomic features and annotated to nearby genes using the annotation pipeline provided by BGI. Genes with nominal *p*-values below 0.05 and absolute values of Δβ ≥ 0.1 in the BGI-supplied DMG output were considered statistically significant and differentially methylated genes. To identify their biological functions, the DMGs were analysed for GO and KEGG enrichment using the ShinyGO 0.85 tool (RRID:SCR_019213) [24], with a false discovery rate below 0.05 and a *p*-value below 0.05 as cut-off values. The WGBS data discussed in this publication have been deposited in NCBI’s Gene Expression Omnibus [25] and are accessible through GEO Series accession number GSE342514.

### 4.5. Statistics and Reproducibility

The statistical analyses used for each experiment are detailed in the figure legends and were calculated using GraphPad Prism version 11.0.2 (RRID:SCR_002798). For comparisons of any two data groups of different samples, *p*-values were calculated using Student’s paired *t*-test. For comparisons of multiple parallel data groups, *p*-values were calculated using one-way ANOVA followed by appropriate post hoc tests for multiple comparisons. Number (*n*) represents the biological replicate, i.e., the individual mouse eye or cell strain. Error bars indicate SD unless otherwise specified. A *p*-value below 0.05 was considered statistically significant: *, *p* < 0.05; **, *p* < 0.01; ***, *p* < 0.001.

## 5. Conclusions

ROCK inhibition by Y39983 suppresses *THBS1* expression and modulates ECM- and cytoskeleton-associated gene expression in primary hTM cells. Targeted MSP suggested a preliminary difference in the unmethylated signal within a primer-defined *THBS1* promoter region, though this observation was not independently supported by a qualifying *THBS1*-associated DMR in the exploratory WGBS analysis. The WGBS analysis generated candidate methylation-associated genes and pathways related to ErbB signalling, focal adhesion, and actin cytoskeleton regulation. However, because multiple-testing correction was not available for the initial DMR/DMG identification, these genome-wide findings are hypothesis-generating rather than statistically validated. Together with the broad effects of 5-azacytidine, the present results suggest a possible association between ROCK inhibition and methylation-related regulation but do not establish a causal DNMT-dependent or DNA methylation-mediated mechanism. Independent validation using quantitative locus-specific assays and appropriately corrected genome-wide analyses is required.

## Data Availability

The WGBS data discussed in this publication have been deposited in NCBI’s Gene Expression Omnibus and are accessible through GEO Series accession number GSE342514. Additional data are available from the corresponding author upon reasonable request.

## Supplementary Materials

The following supporting information can be downloaded at: https://www.mdpi.com/article/10.3390/ijms27209248/s1.

## Abbreviations

5-Aza, 5-azacytidine; TGFβ, transforming growth factor-β; DNMT, DNA methyltransferase; DEX, dexamethasone; ECM, extracellular matrix; hTM, human trabecular meshwork; IOP, intraocular pressure; MSP, methylation-specific PCR; ROCK, Rho-associated protein kinase; THBS1, thrombospondin-1; WGBS, whole-genome bisulphite sequencing; COL4A1, Collagen Type IV Alpha 1 Chain; COL6A1, Collagen Type VI Alpha 1 Chain; ACTG1, Actin Gamma 1; DMG, differentially methylated genes; JCT, juxtacanalicular tissue.
