# Supplementary material for: ROCK Inhibition Suppresses Thrombospondin-1 and Rewires DNA Methylation in the Trabecular Meshwork
Source: Int J Mol Sci. 2026 Oct 18;27(20):9248. doi: 10.3390/ijms27209248 (PMC13705181; doi:10.3390/ijms27209248)
Supplement: Supplementary file 1 [file ijms-27-09248-s001.zip › ijms-4528337-figures.pdf]

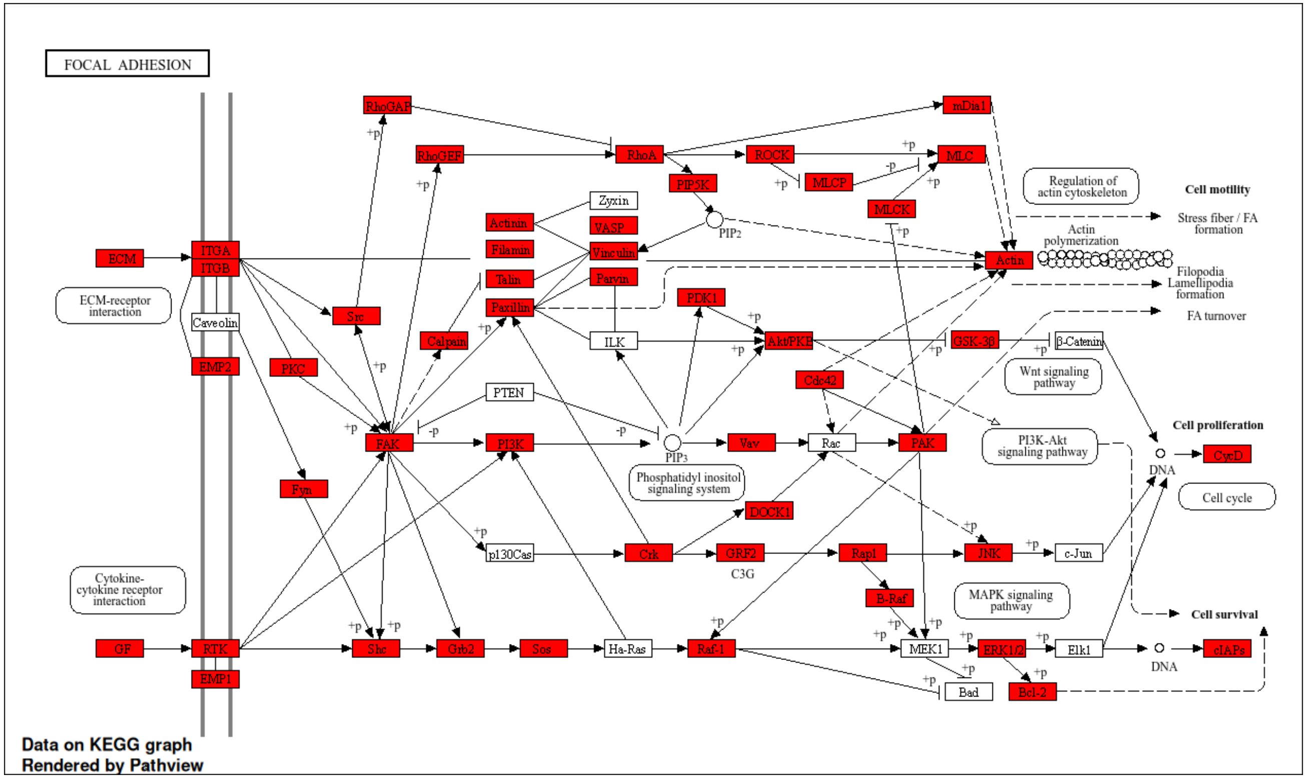

Supplementary Figure S1. Pathview visualization of DMGs mapped to the KEGG focal adhesion pathway in vehicle-control versus Y39983-treated hTM cells. Red nodes indicate genes identified as differentially methylated according to absolute $\Delta\beta \geq 0.1$ and $p < 0.05$.

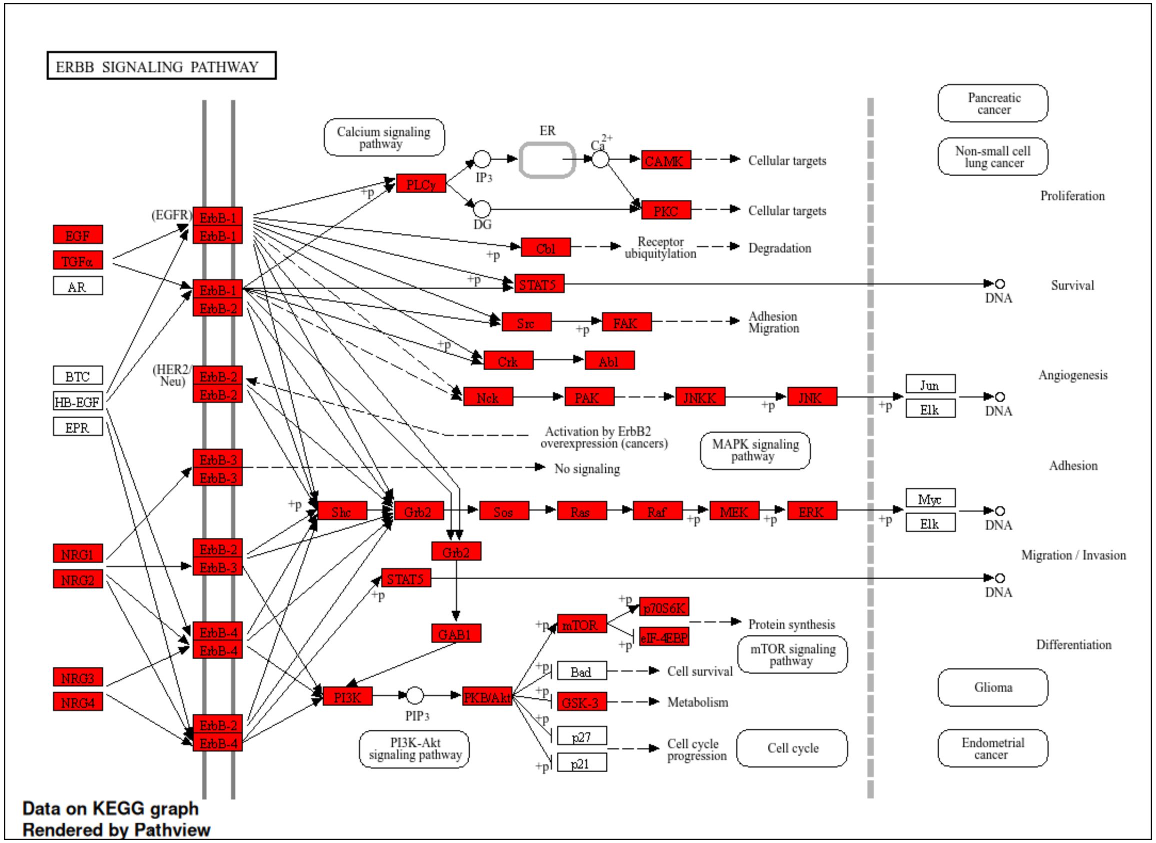

Supplementary Figure S2. Pathview visualization of DMGs mapped to the KEGG ErbB signaling pathway in vehicle-control versus Y39983-treated hTM cells. Red nodes indicate genes identified as differentially methylated according to absolute $\Delta\beta \geq 0.1$ and $p < 0.05$.
